# An *Isospora* Species (Apicomplexa: Eimeriidae) Identified From a Black‐Faced Cuckoo‐Shrike (*Coracina novaehollandiae*) (Gmelin, 1789) (Passeriformes: Campephagidae) in Western Australia

**DOI:** 10.1002/ece3.71298

**Published:** 2025-04-28

**Authors:** Yinhua Chen, Belinda Brice, Bruno P. Berto, Qiong Li, Rongchang Yang

**Affiliations:** ^1^ Maotai Institute Renhuai China; ^2^ Kanyana Wildlife Rehabilitation Centre Lesmurdie Western Australia Australia; ^3^ Departamento de Biologia Animal, Instituto de Ciências Biológicas e da Saúde Universidade Federal Rural Do Rio de Janeiro Seropédica Brazil; ^4^ School of Agricultural Sciences Murdoch University Murdoch Western Australia Australia

**Keywords:** 18S rRNA, 28S rRNA, black‐faced cuckoo‐shrike, Coccidia, COI gene, *Isospora*

## Abstract

We describe and characterize a new *Isospora* species from the black‐faced cuckoo‐shrike ( *Coracina novaehollandiae* ) in Western Australia, using both morphological and molecular approaches. Microscopic analysis of a fecal sample revealed 20 ellipsoidal oocysts, which are subspheroidal to ovoid, measuring 40–43 × 39–41 μm (mean 41.4 × 39.6 μm) with a length/width (L/W) ratio of 1.0–1.1 (mean 1.04). The oocyst wall is bi‐layered (~1.5 μm thick), with a smooth outer layer constituting approximately two thirds of the total thickness. A micropyle is present, characterized by a slight invagination of the inner layer (~6.0 μm wide), but no micropyle cap is observed. Both the oocyst residuum and polar granule are absent. Sporocysts (*n* = 20) are ellipsoidal, measuring 23–24 × 13–14 μm (mean 23.3 × 13.4 μm) with an L/W ratio of 1.7–1.8 (mean 1.74). A flattened to knob‐like Stieda body (~1.5 × 3.0 μm) is present, while sub‐Stieda and para‐Stieda bodies are absent. The sporocyst residuum is composed of granules clustered by a membrane, forming an irregular shape (~12.0 × 8.0 μm). Sporozoites exhibit anterior and posterior refractile bodies and a nucleus. Molecular analyses of the 18S rRNA, 28S rRNA, and COI gene loci demonstrated that this species forms a distinct clade with *Isospora serinuse* in phylogenetic trees based on all three loci. It shares genetic similarities with *I. serinuse* of 98.8%, 93.7%, and 98.9% for the 18S rRNA, 28S rRNA, and COI loci, respectively. Phylogenetic analysis confirms that this new species is closely related to *I. serinuse*. Based on these findings, we propose this isolate as a new species, *Isospora coracinae* n. sp. This is the first coccidian species identified from the Campephagidae family in Australia.

## Graphical abstract

Description of *Isospora* *coracinae* n. sp. from a Black‐faced cuckoo‐shrike *Coracina novaehollandiae.* Morphology study: Unique. Genetic similarities: 98.8%, 93.7%, and 98.9% match to *I. neochmiae* for the 18S rRNA, 28S rRNA, and COI loci, respectively.

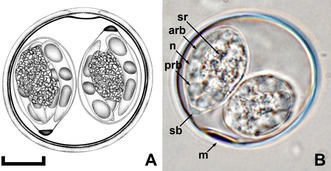

## 1 Introduction

The black‐faced cuckoo‐shrike ( *Coracina novaehollandiae* Gmelin, 1789) is a passerine bird that is widespread in Australia and Tasmania (Taylor and Bonan 2020). The adult bird has a black face and throat, dove‐gray head, white and dove‐gray plumage on its back and wings, and a black tail with a white tip. A hooked black beak is used to feed on a wide variety of invertebrates and insects, fruits, and seeds. It is a member of the Campephagidae family and has been assigned to the *Coracina* genus along with 21 other species (Gill and Donsker 2019). These slender birds often shuffle their wings when they land, which has given rise to them also being called “shufflewings.” They are also known as Gray jays or Cherry hawks (Pizzey and Knight 1997).

The most common coccidia to infect passerine birds are the *Isospora* (Duszynski et al. 1999), with more than 500 species reported (Madani et al. 2018). *Isospora* are members of the Apicomplexa phylum, a group of parasitic protists, belonging to the Eimeriidae family. Many wild and captive birds shed coccidian oocysts in their feces. *Isospora* infections are not commonly associated with causing disease in free‐living birds; however, they may result in severe illness and death in some cases. Although there are many *Isospora* spp. infecting wild birds, only a relatively small number have been both morphologically and genetically characterized (Carreno and Barta 1999; Schrenzel et al. 2005). To date, there has only been one unnamed *Isospora* species reported in the Campephagidae, that from a Pied triller ( *Lalage nigra* Pennant, 1781) by Hegner and Chu (1930).

Taxonomic classification of *Isospora* is becoming more reliant on a combination of both morphological and molecular techniques, including DNA sequencing (Berto et al. 2011).

*Isospora* species that have been both morphologically and genetically described from wild birds in Australia include *Isospora lesouefi* from the endangered regent honeyeater ( *Xanthomyza phrygia* Shaw, 1794), which is endemic to south‐eastern Australia (Morin‐Adeline et al. 2011); *Isospora anthochaerae* from the red wattlebird ( *Anthochaera carunculata* Shaw, 1790) (Yang et al. 2014a); *Isospora streperae* from a gray currawong ( *Strepera versicolor* Latham, 1801) (Yang et al. 2015a); *Isospora manorinae* from a yellow‐throated miner ( *Manorina flavigula wayensis* Gould, 1840) (Yang et al. 2016); *Isospora butcherae* from a silvereye ( *Zosterops lateralis* Latham, 1801) (Yang et al. 2018); *Isospora coronoides* from an Australian raven ( *Corvus coronoides* Vigors and Horsfield, 1827) (Liu et al. 2019); *Isospora lugensae* from a Kerguelen petrel ( *Lugensa brevirostris* Lesson, 1831) (Yang et al. 2021a); *Isospora elliotae* from an Australian magpie ( *Gymnorhina tibicen* Latham, 1801) (Yang et al. 2023) and *Isospora virescensae* from a singing honeyeater (*Gavicalis virescens* Vieillot, 1817) (Chen et al. 2025).

In this study, we describe a species of *Isospora* identified in a black‐faced cuckoo‐shrike from Western Australia and characterize it both morphologically and genetically.

## 2 Materials and Methods

### 2.1 Sample Collection

An adult, black‐faced cuckoo‐shrike was admitted to the Wattle Grove Veterinary Hospital, Perth after it was found by a member of the public with a broken wing. Surgery was performed to pin the fractured humerus. The bird was subsequently transferred to the Kanyana Wildlife Rehabilitation Centre (KWRC) for further treatment and rehabilitation. A fecal sample was obtained soon after admission to KWRC and screened for intestinal parasites.

### 2.2 Morphological Analysis

Numerous embryonated nematode eggs and occasional coccidian oocysts were seen on a wet mount. A small portion of feces was placed in a 2% (w/v) potassium dichromate solution (K_2_Cr_2_ O_7_) and emulsified. This emulsion was stored at 4°C until it was transported to Murdoch University for further study. On arrival at Murdoch University (within 24 h), a thin layer of the emulsion was poured into a Petri dish and stored at room temperature (20°C–22°C), in a dark cupboard. The oocysts were screened daily to assess sporulation, using the 100x oil immersion objective of an Olympus CH‐2 binocular microscope, in combination with an ocular micrometer.

The bird was treated with a broad‐spectrum benzimidazole anthelmintic as well as toltrazuril, an anticoccidial agent, and was successfully released near the found location 6 weeks later.

### 2.3 DNA Isolation

Total DNA was extracted from 200 mg of each fecal sample using the PowerSoil DNA Kit (MolBio, Carlsbad, California) with slight modifications as described by Yang et al. (2016). Specifically, the samples underwent four freeze–thaw cycles, alternating between liquid nitrogen and boiling water, to enhance oocyst lysis before proceeding with the manufacturer's protocol.

### 2.4 PCR Amplification

A nested PCR approach was used to amplify the 18S rRNA gene, 28S rRNA locus, and partial COI gene sequence. For the 18S rRNA gene, primers EiGTF1 (5′‐TTC ACA GGA CCC TCC GAT C) and EiGTR1 (5′‐AAC CAT GGT AAT TCT ATG G) were employed for external amplification, yielding a product of ~1510 bp, while primers EiGTF2 (5′‐TTA CGC CTA CTA GGC ATT CC) and EiGTR2 (5′‐TGA CCT ATC AGC TTT CGA CG) were used for the internal reaction. The PCR reaction mix included 2.5 μL of 10× Kapa PCR buffer, 2 μL of 25 mM MgCl2, 1.0 μL of 10 mM dNTPs, 10 pM of each primer, 1 unit of KapaTaq (Geneworks, Adelaide, SA), 1 μL of DNA (~50 ng) for the external reaction (or 1 μL of external PCR product for the internal reaction), and 16.4 μL of H_2_O. The cycling conditions consisted of an initial denaturation at 94°C for 3 min, followed by 40 cycles of 94°C for 30 s, 55°C for 30 s, and 72°C for 2 min, with a final extension at 72°C for 5 min.

The 28S rRNA locus was amplified using nested PCR with external primers 28SExF (5′‐TAC CCG CTG AAC TTA AGC) and 28SExR (5′‐CMA CCA AGA TCT GCA CTA G), producing a 1362 bp product (Schrenzel et al. 2005). Internal primers 28SInF (5′‐ACT ATG TTC CCT AGT AAC G) and 28SInR (5′‐AAC GCT TCG CCA CGA TCC) generated an amplicon of 1420 bp (Yang et al. 2014a). The reaction mix contained 2.5 μL of 10× Kapa PCR buffer, 2 μL of 25 mM MgCl2, 1 μL of 10 mM dNTPs, 10 pM of each primer, 1 unit of KapaTaq, 1 μL of DNA (~50 ng), and 16.9 μL of H_2_O. Both primary and secondary reactions followed identical cycling conditions: 94°C for 3 min, followed by 35 cycles of 94°C for 30 s, 60°C for 30 s, and 72°C for 90 s, with a final extension at 72°C for 5 min.

The partial COI gene sequence (723 bp) was also amplified using nested PCR with external primers COIF1 (Ogedengbe et al. 2011) and COXR1 (Dolnik et al. 2009) and internal primers COIF2 (Yang et al. 2013) and COXR2 (Dolnik et al. 2009). The reaction mix contained 2.5 μL of 10× Kapa PCR buffer, 2 μL of 25 mM MgCl2, 1.0 μL of 10 mM dNTPs, 10 pM of each primer, 1 unit of KapaTaq, 1 μL of DNA (~50 ng), and 13.4 μL of H_2_O. PCR cycling conditions for both external and internal reactions involved an initial denaturation at 94°C for 3 min, followed by 40 cycles of 94°C for 30 s, 58°C for 30 s, and 72°C for 1 min, concluding with a final extension at 72°C for 5 min.

### 2.5 Sequence Analysisss

Amplicons from the second‐round PCRs were gel‐purified using an in‐house filter tip method as outlined by Yang et al. (2013). The purified PCR products were sequenced in both forward and reverse directions, with duplicate sequencing performed using amplicons from independent PCR runs. Sanger sequencing was conducted using an ABI Prism Dye Terminator Cycle Sequencing Kit (Applied Biosystems, Foster City, California) in accordance with the manufacturer's guidelines.

The sequencing data were analyzed and edited using FinchTV v1.4.0 (http://www.geospiza.com/Products/finchtv.shtml). The resulting sequences were compared to *Isospora* and other coccidian parasite sequences available in GenBank through BLAST searches and aligned with reference sequences using BioEditor (http://bioeditor.sdsc.edu/download.shtml).

### 2.6 Phylogenetic Analysis

Phylogenetic trees for *Isospora* spp. were constructed using partial 18S rDNA, 28S rDNA, and COI gene sequences, aligned with additional isolates retrieved from GenBank. Distance analyses and phylogenetic inferences were performed using MEGA‐X (Barry 2013). Sanger sequencing chromatogram files were imported into MEGA‐X, where the nucleotide sequences for each gene were curated, analyzed, and aligned with reference sequences from GenBank using Clustal W (http://www.clustalw.genome.jp).

Maximum likelihood (ML) trees were generated after determining the most appropriate nucleotide substitution models: TN93 + G + I for 18S and 28S, and GTR + G + I for the COI gene. Bootstrap support values were calculated based on 1000 replicates. Genetic similarities among sequences were also computed using MEGA‐X.

### 2.7 Line Drawing

The line drawings underwent editing using two software applications from CorelDRAW (Corel Draw Graphics Suite, Version 2020, Corel Corporation, Canada), specifically Corel DRAW and Corel PHOTO‐PAINT (Yang et al. 2021a).

## 3 Results

### 3.1 Description of the Oocyst

**FIGURE 1:**
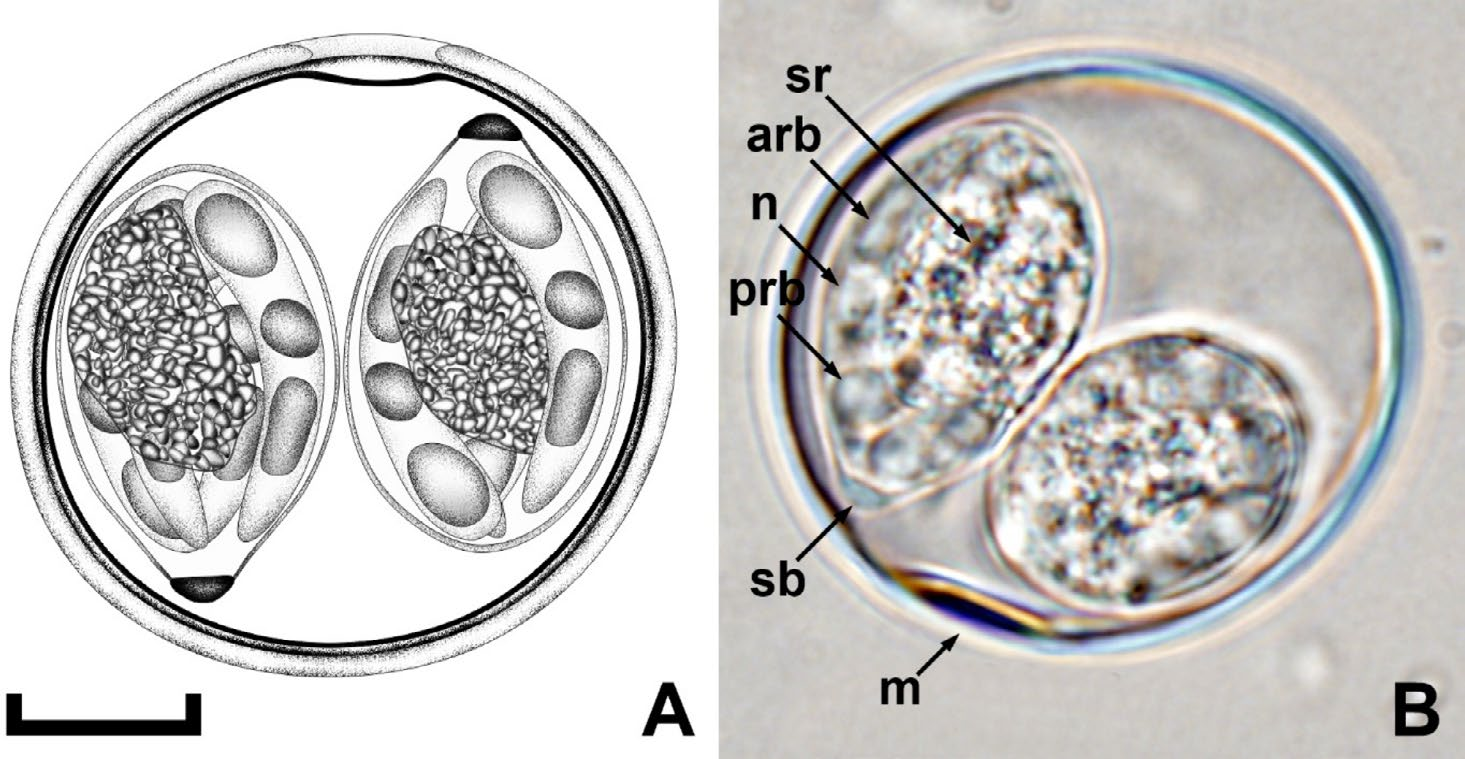
Composite line drawing and photomicrograph of a sporulated oocyst of *I. coracinae* n. sp. from a black‐faced cuckoo‐shrike ( *Coracina novaehollandiae* ) in Western Australia. Note the anterior (arb) and posterior (prb) refractile bodies, micropyle (m), nucleus (n), sporocyst residuum (sr), and Stieda (sb) body. Scale bar: 10 μm.

**FIGURE 2:**
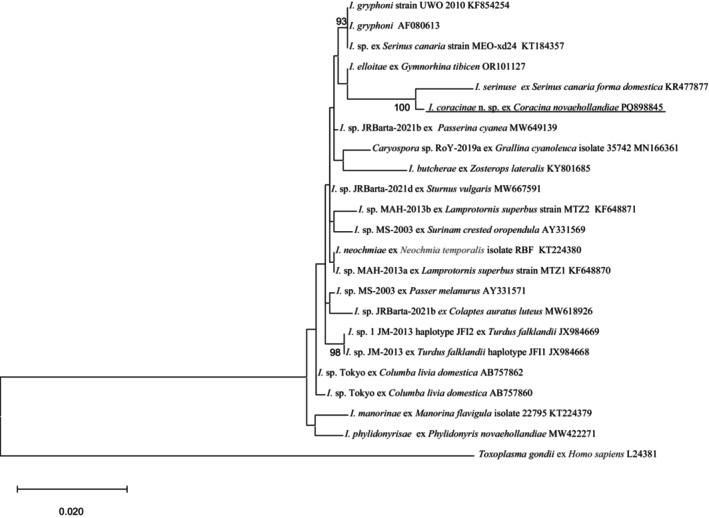
Evolutionary relationships of *I. coracinae n*. sp. inferred by maximum likelihood analysis (ML) of 18S rDNA sequences (1212 bp). Percentage support (> 70%) from 1000 pseudoreplicates from the ML analysis is indicated at the left of the support nodes.

Oocysts (*n* = 20) subspheroidal to ovoidal, 40–43 × 39–41 (41.4 × 39.6); length/width (L/W) ratio 1.0–1.1 (1.04). Wall bi‐layered, ~1.5 thick, outer layer smooth, c. 2/3 of total thickness. Micropyle present with a slight invagination of the inner layer, ~6.0 wide. Micropyle cap absent. Oocyst residuum and polar granule absent. Sporocysts (*n* = 20) ellipsoidal, 23–24 × 13–14 (23.3 × 13.4); length/width (L/W) ratio 1.7–1.8 (1.74). Stieda body present, flattened to knob‐like, ~1.5 × 3.0; sub‐Stieda body and para‐Stieda body absent; sporocyst residuum present, composed of granules clustered by a membrane, in an irregular shape, ~12.0 × 8.0. Sporozoites with anterior and posterior refractile bodies and nucleus (Figures 1 and 2).

Type host: Black‐faced cuckoo‐shrike ( *Coracina novaehollandiae* Gmelin, 1789).

Type locality: Perth (−31.9522°S, 115.8589°E), Western Australia.

Prevalence: 1/1 (100%).

Prepatent period: Unknown.

Patent period: Unknown.

Site of infection: Unknown.

Sporulation time: 48–72 h.

### 3.2 Phylogenetic Analysis

#### 3.2.1 18S rRNA

*Isospora coracinae* n. sp. exhibited 98.8% sequence similarity with *I. serinuse* (GenBank accession: KR477877.2), followed by 98.7% similarity with *I. elliotae* (OR101127), based on pairwise comparisons. As illustrated in Figure 2, *I. coracinae* n. sp. clustered with two *Isospora* species from Western Australia, namely *I. serinuse* and *I. elliotae*, forming a distinct subgroup with *I. serinuse* (Yang et al. 2015b). Pairwise genetic distance analysis between *I. coracinae* n. sp. and 22 reference species/isolates revealed the shortest genetic distance with *I. serinuse* (0.0117), followed by *I. elliotae* (0.0133) (Table S1).

#### 3.2.2 28S rRNA

**FIGURE 3:**
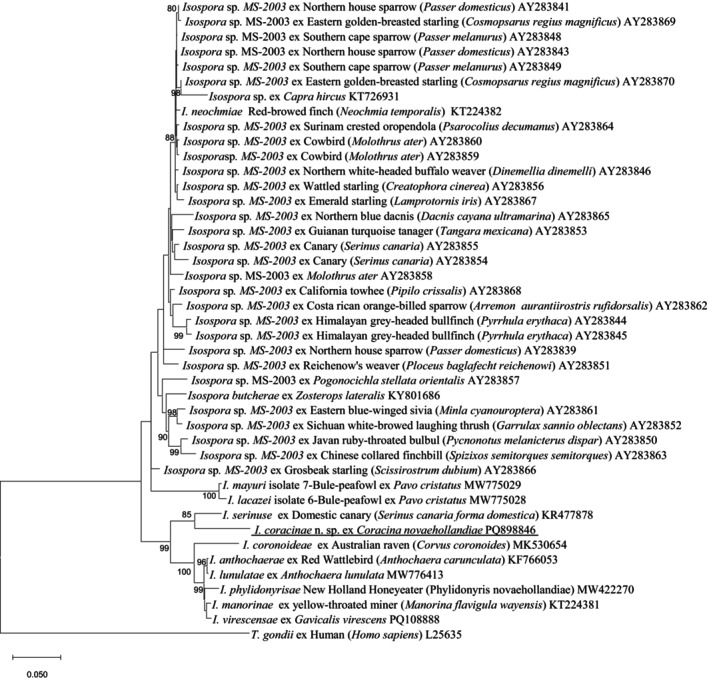
Evolutionary relationships of *I. coracinae* n. sp. inferred by ML of 28S rDNA sequences (1332 bp). Percentage support (> 70%) from 1000 pseudoreplicates from the ML analysis is indicated at the left of the nodes.

*Isospora coracinae* n. sp. exhibited 93.7% sequence similarity with *I. serinuse* (KR477878) based on pairwise comparisons. As shown in Figure 3, *I. coracinae* n. sp. and seven other *Isospora* species identified from passerine birds in WA—namely *I. serinuse* (KR477878), *I. coronoideae* (MK530654), *I. anthochaerae* (KF766053), *I. lunulatae* (MW776413), *I. phylidonyrisae* (MW422270), *I. manorinae* (KT22438), and *I. virescensae* (PQ108888)—clustered into distinct groups. *Isospora coracinae* n. sp. and *I. serinuse* formed a subgroup within a separate clade, which was parallel to the clade comprising the other six *Isospora* species from WA. Furthermore, *I. coracinae* n. sp. showed the shortest genetic distance with *I. serinuse* (0.0627), followed by an unnamed *Isospora* species from a Grosbeak starling in the USA (AY283866), with a genetic distance of 0.0718 (Table S2).

#### 3.2.3 COI Gene

**FIGURE 4:**
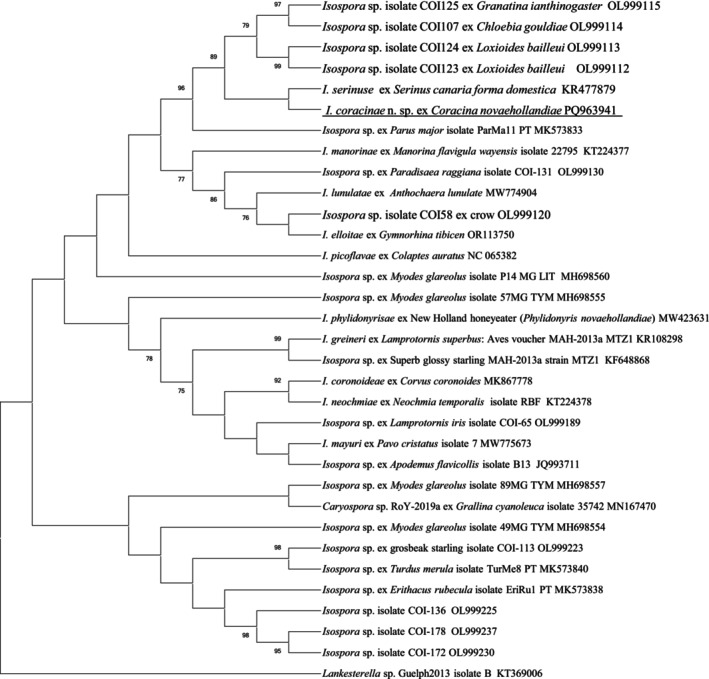
Evolutionary relationships of *I. coracinae* n. sp. inferred by ML of partial *cox1* gene sequences (633 bp). Percentage support (> 70%) from 1000 pseudoreplicates from the ML analysis is indicated at the left of the nodes.

Similar to the results from the 18S and 28S rRNA analyses, *Isospora coracinae* n. sp. exhibited the highest sequence similarity with *I. serinuse* (KR477878) at 98.9%, based on pairwise comparisons. As shown in Figure 4, *I. coracinae* n. sp. and *I. serinuse* formed a subgroup within a distinct clade, which included a pellet subgroup formed by four unnamed *Isospora* isolates from passerine birds in the USA (OL999112 to OL999115). Furthermore, *I. coracinae* n. sp. showed the shortest genetic distance with *I. serinuse* (0.0112), followed by an unnamed *Isospora* species from a palila ( *Loxioides bailleui* ) in the USA (OL999112), with a genetic distance of 0.0227 (Table S3).

## 4 Discussion

Sporulated oocysts of *Isospora coracinae* n. sp. are morphologically distinct from previously characterized *Isospora* species recorded from Passeriformes in Oceania (Trachta et al. 2006; Yang et al. 2014a, 2015a, 2015b, 2015c, 2016, 2021a, 2021b, 2023; Chen et al. 2025). As shown in Table 1, the oocyst dimensions of *I. coracinae* n. sp. (41.4 × 39.6 μm) are larger than those of any previously reported *Isospora* species listed in Table 1.

**TABLE 1:** Morphological comparison of *I. coracinae* n. sp. with other *Isospora* species in the passerine birds.

*Coccidia*	Hosts	References	Oocysts						Sporocysts				
			Shape	Measurements (um)	Shape index	Wall (um)	Polar granule^a^	Oocyst residuum^a^	Shape^b^	Measurements	Stieda body	Substieda body	Residuum
*I. anthochaerae*	*Anthochaera carunculata*	Yang et al. (2014a)	Subspherical	23.4 × 20.7 (20.0–26.0 × 19.0–22.0)	1.1	Bi‐layered c. 0.8	−	−	O	14.5 × 10.1 (11.0–17.0 × 9.0–11.0)	Hemi‐dome	Rectangular‐shaped	Compact
*I. braziliensis*	*Oryzoborus angolensis*	Trachta et al. (2006)	Spherical to subspherical	17.8 × 16.9 (16–19 × 16–18)	1	One‐layered c. 1.0	−	−	E	13.2 × 10.8 (12–14 × 9–12)	Tiny	Absent	Scattered granules
*I. canaria*	*Serinus canaria* Linnaeus	Box (1975), Berto et al. (2011)	Subspherical to ellipsoidal	24.6 × 21.8 (17–30 × 17–30)	1.1	Tri‐layered c. 1.2	+	−	Lemon	18.1 × 11.5 (17.0–22.0 × 1.00–13.0)	Nipple‐like	2.0 × 3.0	Compact
*Isospora coracinae* n. sp.	*Coracina novaehollandiae*	This study	Subspheroidal to ovoidal	41.4 × 39.6 (40–43 × 39–41)	1.04	Bi‐layerd c. 1.5	−	−	E	23.3 × 13.4 (23–24 × 13–14)	Knob‐like	Absent	Scattered granules
*I. curio*	*Oryzoborus angolensis*	Trachta et al. (2006)	Spherical to subspherical	24.6 × 23.6 (22–26 × 22–25)	1	Bi‐layerd c. 1.5	−	−	O	13.2 × 10.9 (15–17 × 10–13)	Small	Absent	Scattered granules
*I. daphnensis*	*Geospiza fortis*	McQuistion (1990)	Ellipsoidal	27.3 × 23.6 (22–30 × 20–27)	1.2	Bi‐layered c. 1.5	+	−	O	15.2 × 10.2 (15.0–16.0 × 9.0–11.0)	Nipple‐like	Small	Scattered granules
*I. elliotae*	*Gymnorhina tibicen*	Yang et al. (2023)	Subspherical	20.7 × 18.7 (19.8–21.6 × 18–19.6)	1.1	Bi‐layered c. 1.5	+	−	O	12.6 × 9.7 (11.9–13.2 × 8.9–10.8)	Flattened to half‐moon	Indistinct	Compact
*I. exigua*	*Camarhynchus parvulus*	McQuistion and Wilson (1988)	Subspheroidal	20.4 × 20.1 (20–23 × 18–23)	1	One‐layered	−	−	O	14 × 9.5 (13–15 × 8–10)	Small	Small	Irregular‐shaped
*I. fragmenta*	*Camarhynchus parvulus*	McQuistion and Wilson (1988)	Subspheroidal	25.3 × 24.2 (24–27 × 23–25)	1	One‐layered	+	−	Piriform	15.4 × 11.5 (14–17 × 11–12)	Knob‐like	Prominent	Irregular‐shaped
*I. gryphoni*	*Carduelis tristis* Linnaeus	Olson et al. (1998)	Spherical	29.2 × 30.7 (25.0–33.0 × 28.0–34.0)	1	Bi‐layered c. 0.8	+	−	O	22.2 × 13.4 (15–25.0 × 12.0–14.5)	Small	Indistinct	Prominent
*I. lugensae*	*Lugensa brevirostris*	Yang et al. (2021a)	Subspherical to ellipsoidal	24.8 × 22.2 (24.0–25.0 × 21.0–23.0)	1.12	Bi‐layered c. 1.0	+	−	O	15.7 × 10.8 (15.0–16.0 × 10.0–11.0)	Knob‐like	Rounded to trapezoidal	Compact
*I. lunulatae*	*Anthochaera lunulata*	Yang et al. (2021c)	Subspheroidal	30.6 × 29.4 (27–34 × 26–31)	1.04	Bi‐layered c. 1.0	+	−	O	18.3 × 10.7 (17–19 × 10–12)	Flattened to rounded	Rounded to rectangular	Compact
*I. manorinae*	*Manorina flavigula obscura*	Yang et al. (2016)	Spherical to subspherical	22.8 × 18.3 (20.3–23.8 × 17.7–18.7)	1.2	Bi‐layered c. 1.3	+	−	Lemon	15.53 × 9.7 (14.6–15.73 × 9.5–9.7)	Hemi‐dome	Rectangular‐shaped	Compact
*I. neochmiae*	*Neochmia temporalis*	Yang et al. (2015c)	Spherical	18.3 × 18.2 (18.2–18.9 × 18.2–18.6)	1	Bi‐layered c. 1.2	+	−	O	13.3 × 8.6 (9.5–16.4 × 6.8–10.0)	Indistinct	Absent	Compact
*I. paranaensis*	*Oryzoborus angolensis*	Trachta et al. (2006)	Subspherical to broadly ellipsoidal	24.3 × 19.8 (22–26 × 18–22)	1.2	One‐layered c. 1.5	+	−	O	15.7 × 10.1 (14–18 × 8–12)	Distinct	Distinct	Spherical
*I. phylidonyrisae*	*Phylidonyris novaehollandiae*	Yang et al. (2021b)	Subspheroidal	29.8 × 29.4 (29–32 × 28–31)	1.01	Bi‐layered c. 1.5	+	−	O	18.4 × 12.3 (18–19 × 12–14)	Flatted	Rounded	Scattered granules
*I. rotunda*	*Camarhynchus parvulus*	McQuistion and Wilson (1988)	Subspheroidal	20.9 × 20.8 (20–24 × 19–23)	1	One‐layered	+	−	O	15 × 9.7 (13–16 × 9–10)	Knob‐like	Prominent	Round
*I. serini*	*Serinus canaria* Linnaeus	Box (1975), Speer and Duszynski (1975)	Spherical to subspherical	20.1 × 19.2 (13.0–23.03 × 13.0–23.0)	1	Tri‐layered c. 1.2	−	−	E	15.2 × 9.4 (13.0–16.0 × 8.0–11.0)	2.0 × 0.6	5.0 × 3.0	Scattered granules
*I. serinuse*	*Serinus canaria* forma *domestica*	Yang et al. (2015b)	Spherical to subspherical	25.5 × 23.5 (24.4–27.0 × 22.0–24.8)	1.09	Bi‐layered c. 1.2	+	−	Lemon	18.9 × 11.8 (17.8–20.2 × 10.6–13.0)	Small	Indistinct	Compact
*I. streperae*	*Strepera versicolor*	Yang et al. (2015a)	Spherical	23.8 × 22.5 (22–24.5 × 21.8 × 24.5)	1.06	Bi‐layered c. 1.0	−	+	O	14.4 × 11.2 (11.5–15.8) × (10.4–12.5)	Hemi‐dome	Rectangular‐shaped	Compact
*I. temeraria*	*Geospiza fortis*	McQuistion and Wilson (1988)	Subspheroidal	25.4 × 21.1 (21–30 × 17–23)	1.2	One‐layered	+	−	Piriform	15 × 10 (14–15 × 9–11)	Knob‐like	Prominent	Round
*I. tristum*	*Acridotheres tristis*	Madani et al. (2018)	Spherical to subspherical	23.3 × 22.3 (18.5–30 × 18.1–29.3)	1.05	Bi‐layered c. 1.3	−	−	O	13.9 × 9.3 (10.2–17.5 × 6.5–12.2)	Flatted	Rounded	Compact
*Isospora virescensae* n. sp.	*Gavicalis virescens*	Chen et al. (2025)	Ellipsoidal	23.4 × 18.7 (21–25 × 18–20)	1.25	Bi‐layered c.1.0	+	−	E	14.1 × 8.7 (14–15 × 8–9)	Flatted	Rounded	Compact

^a^: – = absent,  + = present.

^b^: O = ovoidal, E = elipsoidal.

The comparison of sequences at three loci revealed that *I. coracinae* n. sp. consistently exhibited the highest genomic similarities with *I. serinuse* at the 18S, 28S rRNA, and COI loci, with similarities of 98.8%, 93.7%, and 98.9%, respectively. Phylogenetic analysis also placed *I. coracinae* n. sp. within the same clade as *I. serinuse* across all three loci. However, the grouping of *I. coracinae* n. sp. and *I. serinuse* varied among the three trees, with other *Isospora* species present in surrounding clades. For example, at the 18S rRNA locus, *I. elloitae*, identified in the Australian magpie ( *Gymnorhina tibicen* ), appeared at the base of the clade containing *I. coracinae* n. sp. and *I. serinuse*. Additionally, a subgroup containing three sequences from Canadian birds was observed, including one sequence from *Serinus canaria* (KT184357), identified in a domestic canary, and two sequences from *I. gryphoni* (AF080613 and KF854254). At the 28S rRNA locus, the clade containing *I. coracinae* n. sp. and *I. serinuse* formed a larger group with six additional *Isospora* species identified from passerine birds in Western Australia. At the COI locus, the clade of *I. coracinae* n. sp. and *I. serinuse* grouped with four unnamed *Isospora* isolates (OL999112 to OL999115) identified from passerine birds in the USA.

Many coccidian species, including *Isospora* species, are regarded as being highly host‐specific, typically parasitizing a single host species (Knight et al. 2018). However, exceptions exist, as some coccidian parasites infect multiple host species (Betro et al. 2008; Yang et al. 2014b), even across different genera (McAllister et al. 2024). The host of *I. coracinae* n. sp., the black‐faced cuckoo‐shrike ( *Coracina novaehollandiae* ), is a native bird species in Australia. In contrast, *I. serinuse* parasitizes the domestic canary ( *Serinus canaria* ), a domesticated form of the wild canary. Although the origins of canaries as cage birds remain somewhat unclear, it is believed that they were initially collected from the Canary Islands (Snow et al. 1997).

## 5 Conclusion

Despite the high genomic similarities at the 18S, 28S rRNA, and COI loci between *I. coracinae* n. sp. and *I. serinuse*, their evolutionary paths are entirely distinct. Furthermore, the significant morphological differences in oocysts between the two species support the conclusion that *I. coracinae* is a new *Isospora* species.

## Author Contributions

**Yinhua Chen:** investigation (equal), writing – original draft (equal). **Belinda Brice:** conceptualization (supporting), investigation (equal), methodology (equal), resources (lead), writing – original draft (equal), writing – review and editing (equal). **Bruno P. Berto:** formal analysis (equal), validation (equal). **Qiong Li:** methodology (equal), supervision (equal), writing – review and editing (supporting). **Rongchang Yang:** conceptualization (lead), methodology (equal), supervision (equal), visualization (lead), writing – review and editing (lead).

## Conflicts of Interest

The authors declare no conflicts of interest.

## Supporting information


Data S1.

Data S2.

Data S3.

## Data Availability

The 18S, 28S, and COI additional sequence data generated from this study are accessible at the public domain: https://www.ncbi.nlm.nih.gov/nuccore under the GenBank accession numbers of PQ898845, PQ898846, and PQ963941 for the 18S, 28S, and COI loci, respectively. The 18S, 28S, and COI additional sequence data used in the phylogenetic analysis were derived from the following resources available in the public domain: https://www.ncbi.nlm.nih.gov/nuccore with the GenBank accession numbers in Figures 2, 3, 4.
