# Correction to “An *Isospora* Species (Apicomplexa: Eimeriidae) Identified From a Black‐Faced Cuckoo‐Shrike (*Coracina novaehollandiae*) (Gmelin, 1789) (Passeriformes: Campephagidae) in Western Australia”

**DOI:** 10.1002/ece3.71688

**Published:** 2025-07-01

Chen, Y., Brice, B., Berto, B.P., Li, Q., and Yang, R. (2025) An *Isospora* Species (Apicomplexa: Eimeriidae) Identified From a Black‐Faced Cuckoo‐Shrike (*Coracina novaehollandiae*) (Gmelin, 1789) (Passeriformes: Campephagidae) in Western Australia. *Ecology and Evolution*, 15: e71298. https://doi.org/10.1002/ece3.71298.

On the front page, the affiliation of the first author, Yinhua Chen, and the fourth author, Qiong Li, was listed as “Maotai Institute, Renhuai, Guizhou, China.” The correct affiliation should be “Moutai Institute, Renhuai, Guizhou, China.” In addition, in the Acknowledgement: “This research was partially supported by the Maotai Institute High‐Level Talent Research Launch Fund Project under grant number MYGCCRC2023009” should be corrected as “This research was partially supported by the Moutai Institute High‐Level Talent Research Launch Fund Project under grant number MYGCCRC2023017”.

We apologize for this error.

